# Gene Structure Induced Epigenetic Modifications of *pericarp color1* Alleles of Maize Result in Tissue-Specific Mosaicism

**DOI:** 10.1371/journal.pone.0008231

**Published:** 2009-12-14

**Authors:** Michael L. Robbins, PoHao Wang, Rajandeep S. Sekhon, Surinder Chopra

**Affiliations:** Department of Crop and Soil Sciences, Pennsylvania State University, University Park, Pennsylvania, United States of America; University of Georgia, United States of America

## Abstract

**Background:**

The *pericarp color1* (*p1*) gene encodes for a myb-homologous protein that regulates the biosynthesis of brick-red flavonoid pigments called phlobahpenes. The pattern of pigmentation on the pericarp and cob glumes depends upon the allelic constitution at the *p1* locus. *p1* alleles have unique gene structure and copy number which have been proposed to influence the epigenetic regulation of tissue-specific gene expression. For example, the presence of tandem-repeats has been correlated with the suppression of pericarp pigmentation though a mechanism associated with increased DNA methylation.

**Methodology/Principal Findings:**

Herein, we extensively characterize a *p1* allele called *P1-mosaic* (*P1-mm*) that has mosaic pericarp and light pink or colorless cob glumes pigmentation. Relative to the *P1-wr* (white pericarp and red cob glumes), we show that the tandem repeats of *P1-mm* have a modified gene structure containing a reduced number of repeats. The *P1-mm* has reduced DNA methylation at a distal enhancer and elevated DNA methylation downstream of the transcription start site.

**Conclusions/Significance:**

Mosaic gene expression occurs in many eukaryotes. Herein we use maize *p1* gene as model system to provide further insight about the mechanisms that govern expression mosaicism. We suggest that the gene structure of *P1-mm* is modified in some of its tandem gene repeats. It is known that repeated genes are susceptible to chromatin-mediated regulation of gene expression. We discuss how the modification to the tandem repeats of *P1-mm* may have disrupted the epigenetic mechanisms that stably confer tissue-specific expression.

## Introduction

**Figure 1:**
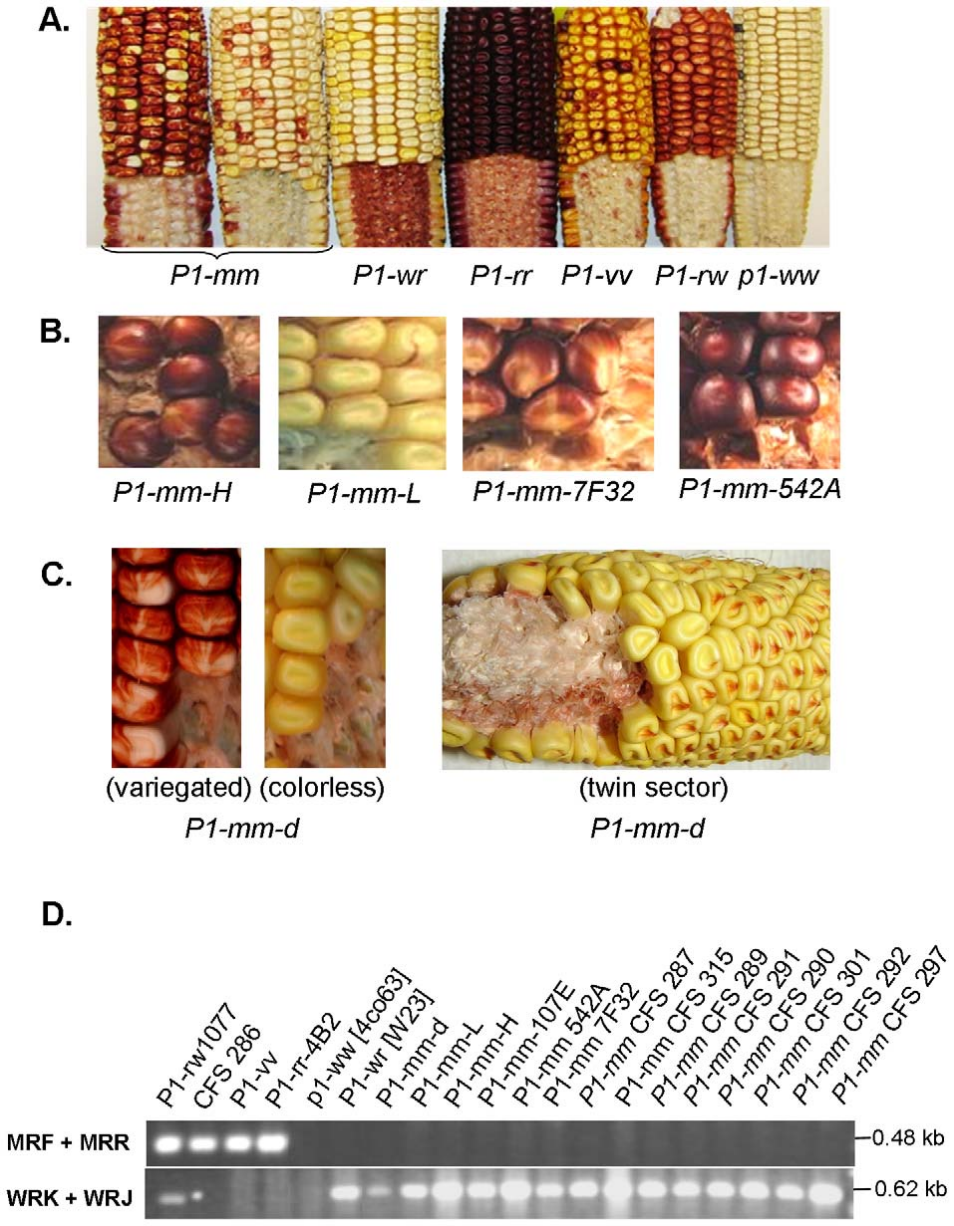
Phenotypic and molecular comparison of *p1* alleles used in this study. **A.** Diverse *p1* alleles conferring different pericarp and cob glume pigmentation patterns. **B.** Representative ears of *P1-mm* stocks that differ with respect to their pigmentation. *P1-mm-H* exhibits a range of heavy variegated pericarp pigmentation and light red cob pigmentation; whereas *P1-mm-L* has light variegated pericarp pigmentation and light pink or colorless cob glume pigmentation. *P1-mm-7F32* and *P1-mm-542A* are self-red revertant stocks of *P1-mm* derived from *P1-mm-H*. **C.** Representative ears from the *P1-mm-d* stock showing several possible pigmentation phenotypes. *P1-wr-d* ears with variegated and colorless pericarp pigmentation are shown. Also shown is an ear that displayed a red cob glumes sector in which the presence of cob pigmentation correlated with a stable “silk scar” type of pigmentation on the pericarp. **D.** PCR analysis to compare *P1-mm* alleles (see Table 1) with previously studied *p1* alleles. Based on the amplification patterns, the *P1-mm* alleles could be classified as molecularly similar to *P1-wr* [W23].

Pericarp pigmentation in maize has important historical relevance in the field of genetics. For example, Gregor Mendel used pericarp pigmentation in maize to verify his work on inheritance ratios in peas [1], [2]. It was in 1869 when Mendel observed the F_2_ segregation from a hybrid cross between two *Zea mays* parents that had colorless and brick-red phlobaphene pigmentation on the pericarp. Decades later, R.A. Emerson showed through genetic linkage analysis that pigmentation on the pericarp and cob glumes was specified by *pericarp color1* (*p1*) [3]. Alleles of *p1* were classified using a two letter suffix system based on their pericarp and cob glumes pigmentation [4]. All stable combinations -*P1-rr*, *P1-wr*, *P1-rw* and *p1-ww* as well as two highly unstable *p1* alleles were described (Figure 1A). The first, *P1-vv*, had variegated pericarp and cob glumes and was initially described by R.A. Emerson in 1914 [5], [6]. The second, *P1-mosaic* (*P1-mm)*, had red mosaic pericarp sectors and colorless or very light cob glumes, and was first described by H.K. Hayes in 1917 [7]. Mosaic pericarp differs from variegated pericarp of *P1-vv* in that boundaries separating red and white regions are often unclear. Consequently, *P1-mm* was considered ‘less satisfactory’ for genetic studies [8]. Although the pigmentation of *P1-mm* is highly unstable, stocks that exhibit different levels of pigmentation including infrequent self-red revertant alleles that resembled the phenotype of *P1-rr* have been maintained [7], [9], [10], [11].

**Table 1:** Description of various *P1-mm* alleles used in this study.

Allele	Origin
*P1-mm-107E*	Unknown
*P1-mm-L*	Maintained light pigmentation though repeated self pollination
*P1-mm-H*	Maintained heavy pigmentation though repeated self pollination
*P1-mm-542A* “RR^a^”	Revertant stock derived from *P1-mm-H*
*P1-mm-7F32* “RR^a^”	Revertant stock derived from *P1-mm-H*
*P1-mm* CFS 287^b^	Unknown
*P1-mm* CFS 315 “RR”a, b	Revertant stock derived from *P1-mm* CFS 287
*P1-mm* CFS 289^b^	Possibly from San Juan, Pueblo, New Mexico
*P1-mm* CFS 291^b^	Unknown
*P1-mm* CFS 290^b^	U.S.A
*P1-mm* CFS 301^b^	Minnesota
*P1-mm* CFS 292^b^	Purchased from a store in Madison, WI
*P1-mm* CFS 297^b^	Tesuque, New Mexico
CFS286^b^	Misclassified as *P1-mm*; has phenotype and gene structure similar to *P1-vv*: Calea, Near Cuzco, Peru

a “RR” denotes that the allele was a stable revertant of *P1-mm* that had red pericarp and red cob glumes pigmentation.

b Alleles' origins are based upon Brink's annotation in [10]. The *P1-mm* CFS 315 allele has been previously called *P1-rr* CFS 315. We report a different name, because it has an identical gene structure as *P1-mm*.

These pioneering maize geneticists recognized the value of flavonoid pigmentation as genetic and phenotypic markers to study the tissue*-*specific expression manifested in the form of alleles [12]. Nearly a century later, the molecular basis for tissue-specific expression of *p1* alleles is becoming clear. The major exception is *P1-mm*, which unlike *P1-vv*, has escaped molecular examination for all these years. The variegated pericarp and cob glumes of *P1-vv* was shown to be attributable to excisions of an *Activator* (*Ac*) transposable element from a *P1-rr* allele [13]. In fact, excisions and reinsertions of *Ac* have generated an allelic series containing insertions throughout the gene [14], [15], [16]. Depending on the location of *Ac*, unique pigmentation patterns on the pericarp and cob glumes were obtained. In one case, the transposition of *Ac* into a distal enhancer of *P1-rr* resulted in the complete loss of cob pigmentation [17]. Accordingly, the lack of cob pigmentation in the endogenous *P1-rw* allele has been attributed to the absence of this enhancer [18].

The basis for the pericarp pigmentation in *P1-wr* was examined by comparing its gene structure and DNA methylation levels with *P1-rr*. The *P1-wr* allele from the W23 inbred line was shown to have six tandemly-arranged gene copies, each containing a proximal promoter and coding sequence that share 99% sequence similarity with the single copy *P1-rr* allele [19]. Moreover, recent sequencing from the B73 inbred line has revealed a *P1-wr* allele with eleven similar tandemly-arranged copies [20]. In both cases, *P1-wr* is hypermethylated as compared with *P1-rr*, however, the presence of a dominant epigenetic modifier *Unstable factor for orange1* (*Ufo1*) leads to a decrease in DNA methylation at *P1-wr*. The reduction in DNA methylation correlates with an increase in ectopic pericarp pigmentation [21].

A critical question pertains to how the copy numbers and structures of *p1* alleles influence their epigenetic states. In order to address this, we set out to identify endogenous alleles that had variations of the copy number of *P1-wr* [W23]. It was suspected that *P1-mm* might have multiple copies because the gain of pericarp pigmentation in *P1-wr* plants in the presence of a dominant modifier *Ufo1* can be variegated or mosaic. Examination of the gene structure revealed that *P1-mm* has a tandem repeat gene structure like *P1-wr*; however, it has a reduced copy number. We show that two gene copies are highly similar to *P1-wr*, while other two copies contain a structurally-distinct region in the second intron. Interestingly, *P1-mm* is also missing the tightly-linked paralogous *pericarp color2* gene, which is located over 100 kb upstream from the *p1* gene [22]. Since it is known that tandemly-repeated sequences are prone to epigenetic gene silencing, we investigated whether *P1-mm* has an altered DNA methylation state. We show here that, as compared to *P1-wr, P1-mm* has a distinct DNA methylation profile; a distal enhancer, shown to be important for pericarp and cob pigmentation [17], [23], [24], [25], is devoid of methylation in *P1-mm*. Conversely, the region immediately downstream from the transcription start site is hypermethylated in *P1-mm*. These opposing DNA methylation modifications suggest that both transcriptional enhancing and suppressing epigenetic mechanisms are responsible for the unique expression patterns of *P1-mm*. The fate of pigmentation would depend on the outcome of competition between these mechanisms.

## Results

### Phenotypic Analysis of *P1-*mosaic

*P1-mosaic* (*P1-mm*) characteristically has a wide range of mosaic pericarp pigmentation and typically has very light or absent cob glumes pigmentation [4], [7], [8], [9], [11], [13], [26]. In agreement with previous studies, we found that *P1-mm* ears may have zones of entirely colorless kernels amongst the red variegated regions. The location of pericarp pigmentation was highly unpredictable and some ears completely lacked pigmentation (Figure 1C). Such an absence of pericarp pigmentation was unstable, as the progeny of ears with colorless pericarp expressed a range of variegated pericarp pigmentation. In the unstable stocks of *P1-mm* a relationship was evident between the pigmentation levels in different tissue types. For instance, the presence of pericarp pigmentation correlated with the presence of silk pigmentation. Similarly, the weak cob glumes pigmentation correlated with a low level of tassel glumes pigmentation. All of these tissues were pigmented in the self-red revertant *P1-mm* stocks (see Materials and Methods and Figure 1B). Interestingly, the *P1-mm-542A* revertant allele also had a striking red pigmentation on the leaf mid-rib that is commonly observed in *P1-rr* alleles. Additionally, the leaf mid-rib pigmentation is also present in some *P1-wr* plants that have been crossed with maize stocks carrying *Ufo1.* Like *P1-wr Ufo1* plants, *P1-mm-542A* plants have a curvature to the stem, suggesting that the aberrant expression of *p1* in leaves may cause pleiotropic developmental defects.

In the highly variegated stock called *P1-mm-d*, we identified some ears that had red sectors on the cob glumes (Figure 1C; see Materials & Methods for stock development). Interestingly, the red cob pigmentation sectors often laid below kernels that had a uniform “silk scar” pigmentation pattern on the pericarps. Thus, the uniform pericarp phenotype correlated with red cob pigmentation. Conversely, variegated or colorless pericarp pigmentation occurred above colorless or very light pink cob glumes. These results suggest that a common mechanism affects both pericarp and cob glumes pigmentation; albeit the suppression of pigmentation in cob glumes is usually more pronounced.

### *P1-mosaic* Resembles *P1-wr*

R.A. Brink and other researchers have collected many different stocks with mosaic pericarp pigmentation (see Materials and Methods) [10]. These mosaic pericarp stocks may have unique origins, or may be similar due to trading amongst researchers or Native American groups [27] prior to entering Brink's collection. Thus, in order to determine if the unique stocks carrying mosaic pericarp have gene structures resembling other *p1* alleles (*P1-rw-1077, P1-rr-4B2, P1-vv, P1-wr* [W23]), we developed an allele-specific PCR genotyping assay. For this assay, the MRF and MRR primer pair was selected because these primers are not present in the *P1-wr* [W23] sequence. In other functional reference *p1* alleles: *P1-vv, P1-rr-4B2*, and *P1-rw1077*, the MRF and MRR primer pair results in a 0.48 kb product (Figure 1D). Additionally, the WRJ and WRK primer pair was chosen because it does not amplify the *P1-vv* and *P1-rr-4B2* alleles. In *P1-wr* [W23] and *P1-rw1077*, WRJ and WRK yields a 0.62 kb band (Figure 1D). Remarkably, all available stocks carrying mosaic pericarp pigmentation (Table 1, Figure 1B–C) were found to have a *P1-wr* [W23]-like PCR amplification pattern (Figure 1D). Additionally, the self-red *“*RR” revertant stocks (*P1-mm-542A, P1-mm-7F32*, and *P1-mm-CFS-315*) derived from mosaic stocks (See Materials and Methods) also exhibited the ‘*P1-wr*’ amplification pattern. Thus, regardless of the origin, all *P1-mm* alleles carried the same PCR-amplification pattern that is present in *P1-wr* [W23].

### *P1-mosaic* and *P1-wr* Have Tandemly-Repeated Gene Structures

**Figure 2:**
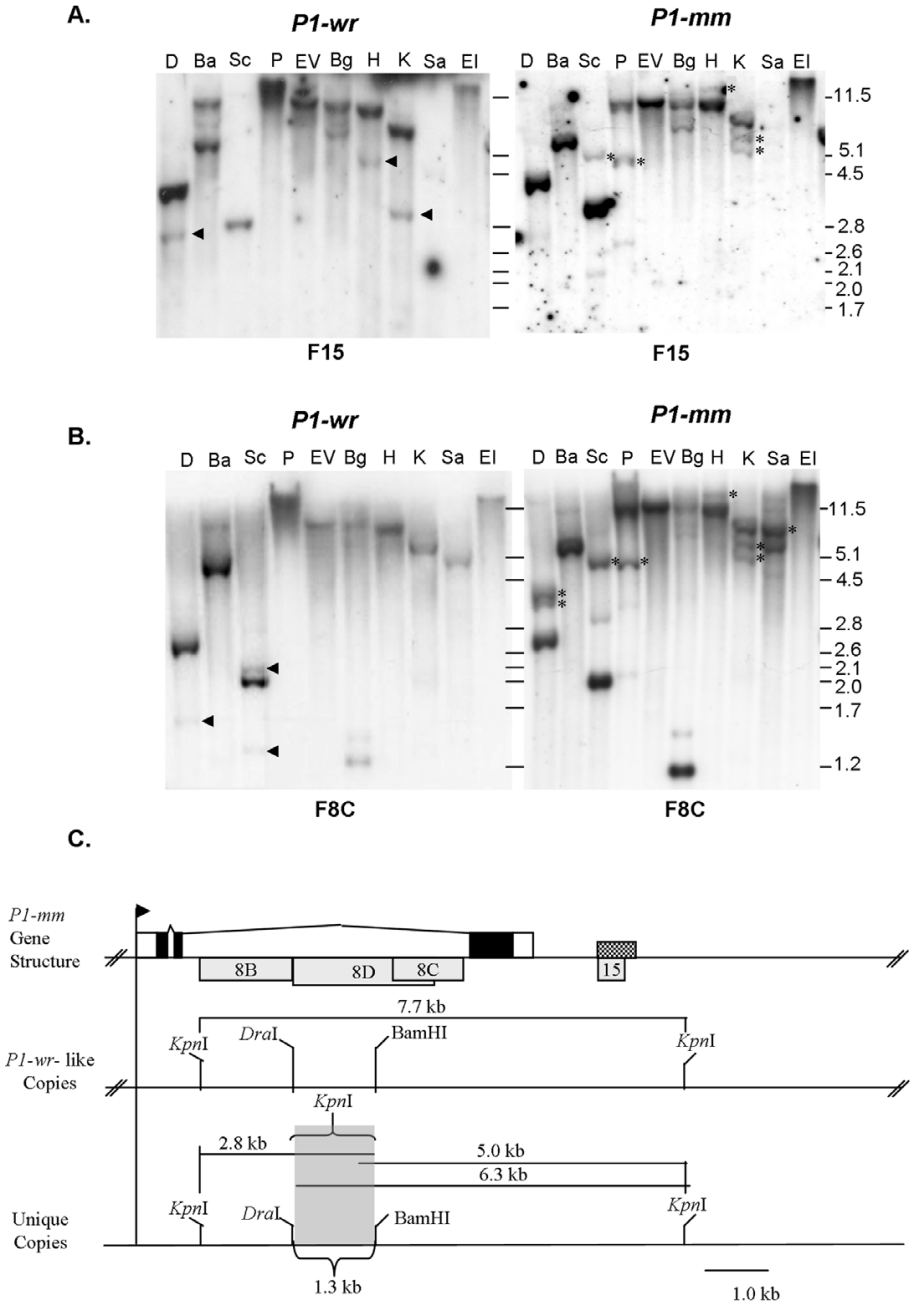
Structural comparison of *P1-mosaic* and *P1-wr* [W23]. Gel blots were prepared by digesting seedling leaf DNA with ten diagnostic restriction enzymes. Enzyme shown are: D, *Dra*I; Ba, *Bam*HI; Sc, *Sca*I; P, *Pst*I; EV, *Eco*RV, Bg, *Bgl*II, H, *Hin*dIII; K, *Kpn*I; Sa, *Sac*I; EI, *Eco*RI. Blots were hybridized with *p1* probes corresponding to the distal floral organ enhancer F15 (**A**) and intron 2 fragment F8C (**B**). The sizes of molecular weight marker bands are indicated in kilobase pairs to the right of blots. **C.** The *P1-mm* gene structure diagram is based upon the published sequence of *P1-wr* [W23] (Accession EF165349). The positions of exons are shown as rectangles and introns are represented by the connecting lines. The shaded regions of the rectangles represent coding sequence, whereas the unshaded regions represent the UTRs. The arrow represents the transcription start site. The hash marks indicate the positions of linked copies in the tandem gene array. The checkered box shows the position of the *p1* distal enhancer, which is present in every gene copy. *p1* probe fragments are indicated below the gene structure diagram as grey rectangles. Shown below the map of *P1-wr* are gene copies of *P1-mm* that are structurally similar (top) and unique (bottom) from *P1-wr*. The grey shaded box indicates the region of these copies that is shown to be structurally unique. The sizes of *Kpn*I fragments are given in kilobase pairs.

The PCR genotyping assay allowed us to ascertain sequence similarity between *P1-mm* and *P1-wr*. In order to explain the marked phenotypic differences between these two alleles, we further compared their gene structures by DNA gel blot analysis. Diagnostic bands revealed that *P1-mm* shares a tandem-repeat gene structure with *P1-wr* [W23] (Figure 2) [19]. For example, a 12.6 kb *Eco*RV band results from the cleavage of intron 2 *Eco*RV sites in adjacent copies. To verify the extent of the similarity between *P1-mm* and *P1-wr,* we sequenced the 5′ and 3′ ends of cDNA from three *P1-mm* alleles (*P1-mm-H, P1-mm-L*, and *P1-mm-542A*) (Figure S1). There was a perfect match to *P1-wr* [W23] sequence at the 5′ end and near perfect match at the 3′ end (We detected a possible base substitution for the *P1-mm-542A* allele at the 3′ end). Conversely, the sequence of *P1-rr-4B2* is clearly unique at both regions, indicating that *P1-wr* and *P1-mm* indeed have common lineages. Though most of the *P1-mm* promoter remains to be sequenced, limited sequence data was generated while testing the DNA methylation for a 439 bp sequence at the *p1* distal enhancer (genomic bisulfite sequencing data is presented later). Interestingly there was a 4 bp insertion in *P1-mm* (after position 1198 of *P1-wr* accession EF165349) in the distal enhancer of all 60 *P1-mm* clones analyzed. Since the multicopy *P1-wr* [W23] allele did not have this insertion, we may infer that *P1-mm* and *P1-wr* could have diverged from a common ancestor and underwent separate tandem duplication events. Alternatively, a recombination-based mechanism may have led to the presence of the 4 bp insertion in every *P1-mm* gene copy after a common tandem duplication event. All other *p1* alleles with available sequences (*P1-rr, P1-rw* and *P1-wr*) also do not have the 4 bp insertion, making it unique to the sequence of *P1-mm*.

### *P1-mosaic* Is Missing Sequence Present in *P1-wr* and the Linked Paralogous *pericarp color2* Gene

There are important differences between the gene structures of *P1-mm* and *P1-wr*, as indicated by other bands in Figure 2. Specific bands detected in *P1-wr*, were absent in *P1-mm* (see bands marked with arrowheads). For example, the distal floral organ enhancer probe fragment 15 did not detect 4.6 *Hin*dIII, 3.1 kb *Kpn*I, 2.8 kb *Dra*I and 1 kb-*Sal*I fragments in *P1-mm* (Figure 2A). A 3.0 kb *Sal*I band that is homologous with intron 2 probe 8B of *P1-wr* [W23] was also not detected in *P1-mm* (M. Robbins and S. Chopra, unpublished). Additionally, the *p1* intron 2 probe F8C did not detect 2.1 and 1.3 kb *Sca*I bands or a 1.68 kb *Dra*I band (Figure 2B). The 1.3 kb *Sca*I band and the 1.68 kb *Dra*I band are derived from the linked *pericarp color2* (*p2*) gene (Accession number AF210616.1), which is paralogous with *p1. p2* is located over 100 kb upstream from the *p1* gene and is expressed specifically in silk tissue along with *p1* to yield a brownish pigmentation [22]. Since *p2* is not present in *P1-mm*, the presence of silk pigmentation would depend solely on the level of *p1* expression.

### *P1-mosaic* Has Structurally Unique Copies Containing a Diverse Intron 2 Region

The absence of *P1-wr*-hybridizing sequence from *P1-mm* indicated that considerable structural differences exist between their respective gene structures. The region of the tandem array of *P1-mm* that is most structurally unique could be discerned based on the presence of several *P1-mm*-specific bands (indicated with asterisks on Figure 2). Particularly with *Dra*I and *Bam*HI digestions, the polymorphic region could be inferred. With intron 2 probe F8C, *Dra*I produces two unique bands of approximately 3.8 and 4.0 kb size in *P1-mm* (Figure 2B). However, the *Bam*HI banding pattern detected with probes F8C and F15 was similar in *P1-wr* and *P1-mm* (Figure 2 and data not shown). These results indicate that structural difference in *P1-mm* reside within a 1.3 kb region downstream from a *Dra*I site and upstream from a *Bam*HI in the large (4.6 kb) intron 2 (see grey-shaded box in Figure 2C). Gel blots carrying *Kpn*I digested genomic DNA also indicate that there are structural differences at this location. For example, in *P1-wr* [W23], probes F15, F8B, and F8C are all contained within the same 7.7 kb *Kpn*I fragment. However, in *P1-mm*, additional *Kpn*I bands were observed: fragments F8C and F15 detect two bands of approximately 5.0 and 6.3 kb, whereas fragment F8B detects a single 2.8 kb band (Figure 2). This suggests that *Kpn*I site(s) separate intron 2 fragments 8B and 8C in two unique *P1-mm* copies. One likely scenario is that these unique *P1-mm* copies could contain end deletions of the *p1* tandem gene array. The absence of the upstream-linked *p2* gene in *P1-mm* could be explained by an extensive deletion that also affects the 5′ end of the *p1* tandem gene array. However, to accurately assemble the structure of the unique *P1-mm* copies, their sequencing will be required. It is important here to re-emphasize that the expected band sizes found in the tandem array of *P1-wr* are also found in *P1-mm*, meaning that some copies are structurally similar to *P1-wr*.

### *P1-mosaic* Exhibits a Reduced Copy Number Relative to *P1-wr*

**Figure 3:**
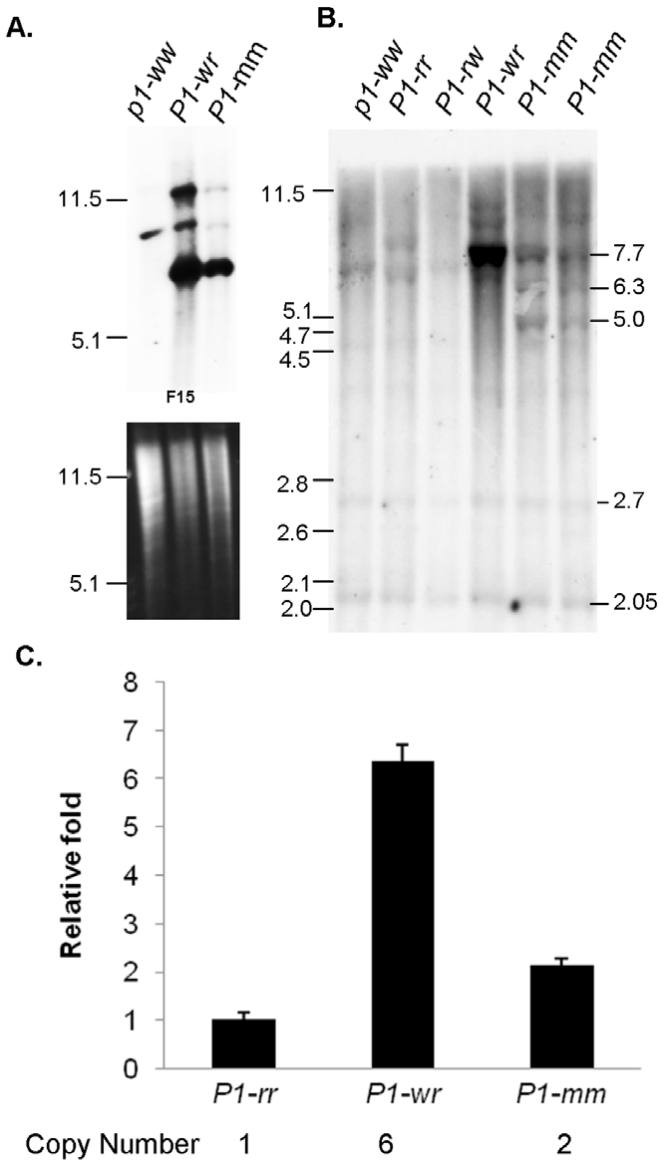
DNA gel blot analysis showing the copy number and structure of *P1-mm*. **A.** The copy number was inferred from *Bam*HI digested DNA gel blots hybridized with the *p1* distal floral organ enhancer fragment F15. The ethidium bromide stained gel indicates the relative DNA loading. Lanes are shown with names of alleles. **B.** Copy number was inferred from *Kpn*I digested DNA gel blots hybridized with intron 2 probe fragment F8C. Molecular weight marker band sizes are shown to the left of the blots. The sizes of bands discussed in the text are indicated in kilobase pairs to the right of the blot. **C.** Real-time quantitative PCR was performed to estimate the copy number using *P1-rr*-*4B2* as reference genotype (single copy). The relative fold is calculated using *P1-rr*-*4B2* as a reference. Vertical lines indicate standard error (n = 3).

The missing *p1* homologous bands in *P1-mm* indicated that some of the *P1-wr* gene structure is absent from *P1-mm*. Consequently, we considered the possibility that *P1-mm* may have a reduced copy number in its tandem gene array. To test this hypothesis, *P1-wr* [W23] and *P1-mm* genomic DNA were digested with *Bam*HI and hybridized with the distal floral organ enhancer probe fragment 15. The signal intensity achieved from *P1-mm* was considerably less when compared with *P1-wr* [W23], indicating that the copy number of *P1-mm* was significantly lower than that of *P1-wr* (Figure 3A). The relative copy number of *P1-mm* and other *p1* alleles was also compared on a gel blot carrying *Kpn*I-digested DNA which was hybridized with intron 2 probe F8C (Figure 3B). The 2.7 and 2.05 kb bands can be considered as internal DNA loading controls, showing that all lanes had equal loading except the *P1-rw* lane, which has a reduced DNA loading. We inferred that the signal intensity in *P1-mm* of an expected 7.7 kb band was much reduced, as compared with *P1-wr* [W23]. However, the intensity was only slightly greater than the signal derived from the single copy *P1-rr-4B2* allele. Consequently, we estimated that *P1-mm* has two intact gene copies. The *Kpn*I restriction bands of 5.0 and 6.3 kb present in *P1-mm* and absent from *P1-wr* are derived from the two unique *P1-mm* gene copies. To verify the estimation of the copy number in *P1-mm* from Southern blot hybridization, real-time quantitative PCR (RT-qPCR) was performed using primers specific to *p1* intron II probe F8B. *P1-rr-4B2* was used as a reference genotype for single copy number. The RT-qPCR result showed a perfect match with Southern blot hybridization: *P1-mm* and *P1-wr* [W23] were identified to have two and six copies respectively (Figure 3C). This result also confirms the previous Southern blot-based estimation of *P1-wr* [W23] copy number [7].

### Diverse *P1-mosiac* Alleles Have Identical Gene Structures

The *p1* gene structures for all *P1-mosaic* stocks in Table 1 were compared with one another. Diagnostic digestion with *Kpn*I was used to analyze the alleles from R.A. Brink's collection using *p1* probes 8C (intron II) and F15 (distal enhancer) [10]. The structures of other accessions (*P1-mm-107E, P1-mm-d, P1-mm-H, P1-mm-L, P1-mm-7F32*, and *P1-mm-542A*) were determined with different restriction enzymes. Interestingly, the structures of all *P1-mm* alleles were found to be identical. For simplicity sake, only the gene structure of one *P1-mm* allele is shown (Figure 2). Importantly, the stable self-red (i.e. “RR”) revertant alleles of *P1-mm* had the same gene structures as the unstable variegated alleles. This result strongly suggests that the revertant alleles did not arise by germinal transposon excisions from unstable *P1-mm* alleles. Therefore, transposon excision from *P1-mm* is not likely the cause of the mosaic pericarp pigmentation.

### Major Maize Transposon Families Are Not Involved in the Regulation of *P1-mm*

In addition to the aforementioned gene structure analysis, several genetic tests support the assertion that the transposase activity of major maize transposon families is not involved in the regulation of *P1-mm* Crosses of *P1-mm* with an *Ac-* tester stock have determined that active *Ac* element(s) are not responsible for the phenotype of *P1-mm* [13]. Subsequently, it was reported that mosaic pericarp is still possible in the absence of active *Spm* elements [28], [29], [30]. A major class of maize transposable elements that had not been tested with respect to pericarp pigmentation of *P1-mm* belongs to the *Mutator (Mu)* family. In order to silence *Mu* elements, we crossed a homozygous *P1-mm* stock with a *p1-ww* stock heterozygous for *Muk*. *Muk* encodes an inverted repeat transcript that functions to silence the *MuDR* transposase which is required for the mobility of *Mu* elements [31], [32], [33]. F_1_ individuals were genotyped for the presence of *Muk* by PCR (see Methods). The range of mosaic pericarp pigmentation was similar for the 12 *Muk* and 12 wild type plants assayed, indicating that *Mu* activity does not affect the *P1-mm* phenotype (not shown).

### DNA Methylation of *P1-*Mosaic Suggests the Involvement of an Epigenetic Mechanism

**Figure 4:**
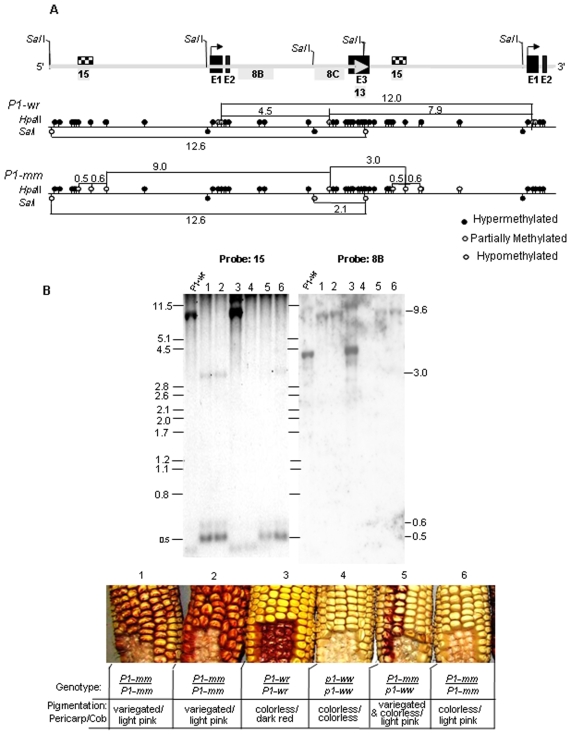
*P1-mosaic* has a highly unique DNA methylation pattern as compared to *P1-wr*. For this analysis, the *P1-mm-d* allele was used. **A.** *P1-wr* [W23] was used as a template [19] to construct the DNA methylation map for *P1-mm*. The intron/exon structure of *P1-wr* is shown as a line diagram above the methylation maps. The large grey arrow shown on the line diagram represents the end of a copy in the tandem array. The bent arrow indicates the location of the transcription start site. Exons are abbreviated as E1, E2, and E3. The placement of *p1* probes (grey shaded boxes) is shown immediately below the line diagram. DNA methylation maps are shown below the gene structure. On the DNA methylation maps, black circles indicate hypermethylated sites; grey circles indicate partially-methylated sites; non shaded circles represent hypomethylated sites. **B.** DNA methylation analysis of *P1-mosaic* sibling plants differing for pericarp pigmentation. Seedling leaf genomic DNA from plants of indicated genotypes was digested with the methylation-sensitive restriction enzyme *Hpa*II and the resulting blot was sequentially hybridized with *p1* probes corresponding to the distal floral enhancer fragment F15 and intron 2 fragment F8B. Ear phenotypes from plants used for DNA methylation analysis are shown below the blots. Genotype and phenotype information is also provided in the table below each ear. The methylation map showing *P1-mm* in **A** was constructed based on the results of these hybridizations. Since some *P1-mm* copies are structurally unique from *P1-wr* at a region in intron 2 (see Figure 2), the placement of fragments which span probe F8C were estimated based on the published sequence of *P1-wr* [W23].

We showed that *P1-mm* alleles resemble the tandem-arrayed gene structure of *P1-wr*, albeit with structural differences in intron 2 in some copies. Additionally, we showed that the *P1-mm* has two rather than the six copies present in *P1-wr* [W23]. Given that *P1-wr* is hypermethylated relative to the single copy *P1-rr* allele [19], we considered the hypothesis that reduced copy number contributes to the presence of pericarp pigmentation. DNA gel blot analysis was used to construct a DNA methylation map comparing *P1-mm* and *P1-wr* [W23] (Figure 4A). With *Sal*I, *P1-wr* [W23] produces a 12.6 kb band that extends the entire length of the gene. This is because two of the three *Sal*I sites in *P1-wr* [W23] are hypermethylated. Interestingly, in *P1-mm* we detected an additional 2.1 kb band, which suggested that a *Sal*I site in intron 2 (at position 10,310 of accession EF165349) was partially hypomethylated (Figure 4A). Considerable hypomethylation was also detected at the distal floral organ enhancer, as evidenced by diagnostic 500 bp and 600 bp F15-homolgous *Hpa*II bands (Figure 4A, B). The presence of these small molecular weight bands in *P1-mm* was correlated with the complete absence of a 7.9 kb *Hpa*II band. This result demonstrated that *P1-mm* copies are hypomethylated at the single *Hpa*II site contained within the distal enhancer region. However, the presence of a probe F15 hybridizing 3.0 kb *Hpa*II band in *P1-mm* suggests that the DNA methylation might be similar to *P1-wr* [W23] upstream from this distal enhancer (Figure 4A).

**Figure 5:**
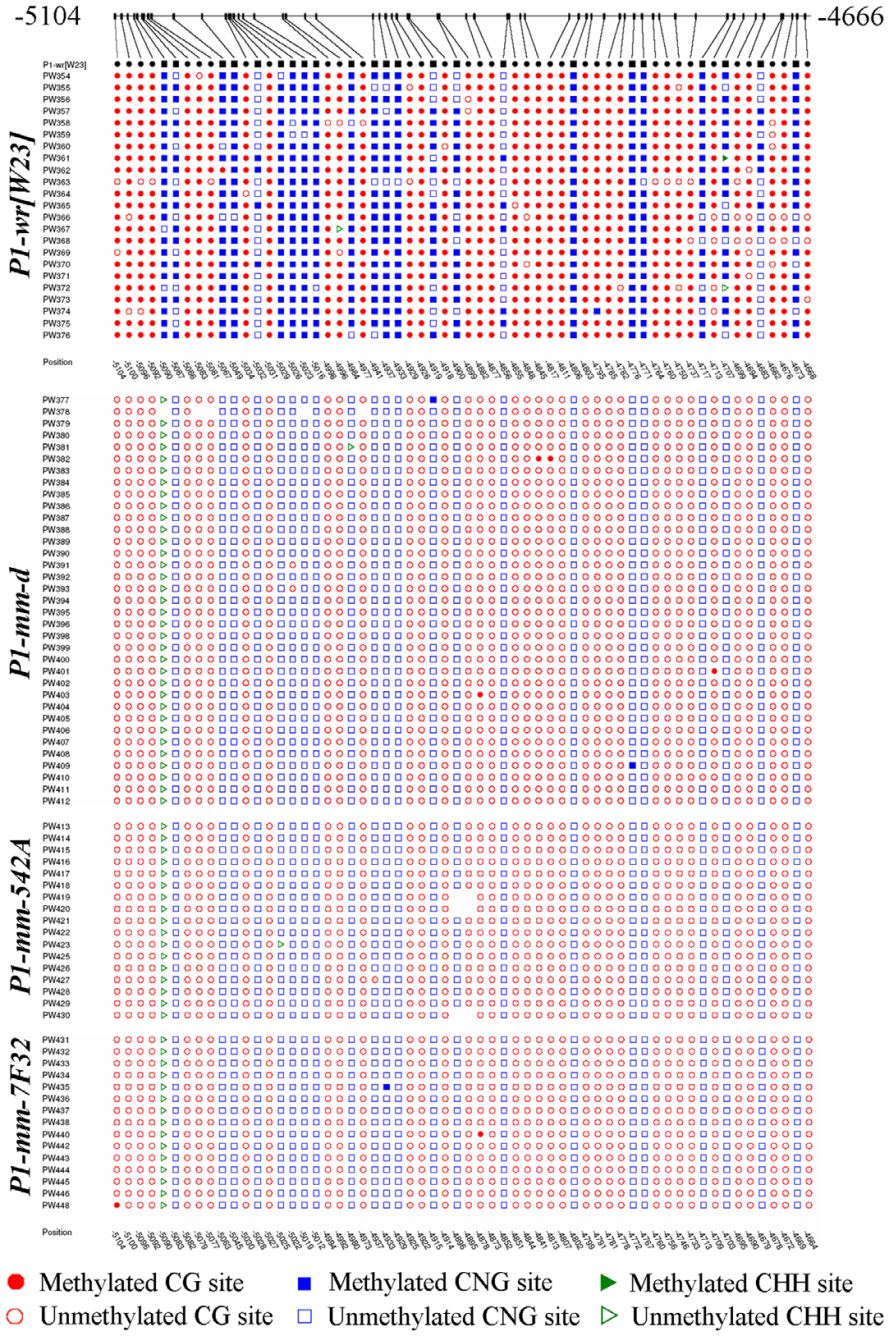
The distal floral enhancer of mosaic and self-red revertant alleles of *P1-mm* has negligible levels of DNA methylation when compared to *P1-wr* [W23]. The DNA methylation of a 439 bp fragment located at the 3′ end of a distal floral organ enhancer was analyzed by genomic bisulfite sequencing. The length of this fragment is 443 bp for *P1-mm* alleles due to a 4 bp insertion (See Materials and Methods for details). The location of this distal enhancer is shown as a checkered box on the gene structure diagram in Figure 4A. Seedling leaf genomic DNA from *P1-*wr [W23], *P1-mm-d* and the self-red revertant alleles *P1-mm-7F32* and *P1-mm-542A* was used for this assay. Methylation status of cytosines in CG (circles) and CNG (squares) context in individual clones of the *p1* alleles is shown. One triangle present in three *P1-mm* alleles represents a CNN site that was created due to the 4 bp insertion in this region. The rest of the CNN sites are not shown as they completely lacked methylation in *P1-wr* [W23] and the three *P1-mm* alleles. [50]

To further resolve methylation status of individual cytosine residues, we performed genomic bisulfite sequencing of a 439 bp region of the *p1* distal enhancer (positions -5104 to -4666 of EF165349) (Figure 5). This region has been previously shown to house enhancer elements that are important for *p1*-induced pericarp and cob pigmentation [21], [34]. In agreement with our previous observations (Chopra 2003; Sekhon 2007), *P1-wr* [W23] has high levels of CG and CNG methylation in the region as indicated by predominantly filled circles and squares, respectively. Interestingly, the distal enhancer region of mosaic and self-red revertant *P1-mm* alleles was essentially devoid of DNA methylation in CG and CNG contexts. Such an absence of DNA methylation at the distal enhancer region was also observed for *P1-rr*, which shows uniformly red pericarp and cob pigmentation (R. Sekhon and S. Chopra, unpublished). Finally, CNN methylation levels in the distal enhancer region were negligible for all the alleles tested (data not shown). Overall, these results show that the *P1-mm* alleles are uniformly hypomethylated and that the mosaic (*P1-mm-d*) and self-red reverent (*P1-mm-7F32* and *P1-mm-542A*) alleles do not differ for their methylation across the distal enhancer region.

As compared with *P1-wr, P1-mm* has a striking increase in DNA methylation downstream from the transcription start site (Figure 4A). This is evidenced by the absence of a 4.5 kb *Hpa*II band that is homologous with intron 2 probe fragment 8B (Figure 4B). The 4.5 kb band size results from digestion of a *Hpa*II site downstream from the transcription start. In *P1-mm* the 4.5 kb band is replaced by a 9 kb fragment (Figure 4B). The 9 kb fragment can be explained by increased DNA methylation at *Hpa*II site(s) downstream from the transcription start site (Figure 4A). The 9 kb band size also takes into account the absence of DNA methylation at the distal enhancer, as previously discussed. The F8C probe region of intron 2 in *P1-mm* contains a large number of low intensity *Hpa*II bands that were not placed on the methylation map (M. Robbins and S. Chopra unpublished). This result was expected, since the F8C probe is adjacent to the region where we have identified structural differences between *P1-mm* and *P1-wr* (see Figure 2C). The *Hpa*II sites producing these bands are predicted to reside in the structurally unique copies of *P1-mm*. We found that the intron 2 region, like the distal enhancer region, exhibited no difference in the *Hpa*II banding pattern between mosaic and revertant *P1-mm* stocks using *p1* probe F8C.

In summary, DNA methylation did not correlate with establishment or maintenance of pigmentation levels in *P1-mm* and was thus not predictive of the level of phlobaphene pigmentation. The same DNA methylation pattern was present for individuals with variegated, colorless, and self-red revertant pericarp (Figure 4B). It is, however, important to stress that altered DNA methylation in *P1-mm*, as compared with *P1-wr* [W23], is predictive of modified chromatin packaging [35]. A highly interesting pattern was observed: *P1-mm* is devoid of methylation at a distal floral enhancer and is hypermethylated downstream from the transcription start site.

## Discussion

### Phlobaphene Pigmentation Is Correlated with the Copy Number of *p1* Alleles

Tandem repeats have been linked with heterochromatin-associated gene silencing [36]. Transgenes that incorporate as arrays of multiple copies are especially prone to variegation associated with their silencing [37]. Such Repeat Induced Gene Silencing (RIGS) of transgenes in plants involves a homologous DNA pairing mechanism that correlates with DNA methylation levels [38]. The pairing of homologous DNA in ‘RIGS’ parallels that described for Position Effect Variegation (PEV) in *Drosophila* [39]. In PEV, the variable placement of a marker gene in euchromatin or heterochromatin dictates its expression [40]. Positioning in heterochromatin is enhanced by the presence of multiple gene copies, and an increased level of variegated silencing is thought to involve pairing interactions between copies [41], [42], [43], [44]. Such preferential pairing interactions between homologous transgenes have also been observed in *Arabidopsis* by fluorescent chromatin tagging experiments [45]. Pairing may be accomplished by proteins binding to identical sites in linked copies, which subsequently form homodimers that strengthen chromatin association [36], [38], [39], [46]. In *P1-wr*, the multiple tandemly-arranged copies may similarly associate to direct heterochromatin formation. Evidence that supports this copy paring hypothesis in *P1-wr* was presented for a *Mu* transposon insertion allele called *P1-wr-mum6* that contains a *Mu1* transposon in the 5′UTR of one of the *P1-wr* gene copies [47]. *P1-wr-mum6* exhibits ectopic pericarp pigmentation that was shown to arise from the increased expression of gene copies that were not interrupted by the transposon. Furthermore, the stable gain of pericarp expression in *P1-wr-mum6* correlated with the hypomethylation of a distal floral organ enhancer. Thus, disruption of a single *P1-wr* gene copy can lead to the loss of suppression of other wildtype copies, which suggests for a possible association between these gene copies.

In opposition to longstanding dogma in the *P1-mm* literature, our genetic and molecular data suggest that the characteristic mosaic pigmentation of *P1-mm* arises by a mechanism that does not involve active transposon insertions or excisions. Rather, the unique gene structure of *P1-mm* itself may influence its variable expression states. We showed that both *P1-mm* and *P1-wr* have tandemly-repeated gene copies; however, *P1-mm* had a significantly lower copy number and was missing *p1* homologous sequences that are predicted to be linked. Interestingly, we found that the upstream linked *p2* gene which, is a paralog of *p1* that is exclusively expressed in silk [22], is not present in *P1-mm*. The absence of *p2* suggests a deletion 5′ at the *p1* gene in *P1-mm*. Such deletion(s) may have also affected the tandem array of *P1-mm* since two gene copies of *P1-mm* were found to be structurally unique from *P1-wr* at an intron 2 region. These unique copies in *P1-mm* may be truncated versions of previously functional copies that could have been required for stable tissue-specific expression patterns. It is possible that the unique *P1-mm* copies could be a source of aberrant transcripts that initiate siRNA-based silencing in a variable fashion to yield the mosaic pigmentation patterns. For example, a truncated copy of the tandemly repeated barley *Mlo* gene produces siRNA that functions to block the expression from wild type copies [48].

The location of the structural difference in the intron 2 region of *P1-mm* may be critical for governing its phenotype. Importantly, the intron 2 region contains a 168 bp regulatory sequence that has been implicated in the specification of cob pigmentation in *P1-wr* [49]. The DNA hypermethylation of this 168 bp sequence was correlated with the loss of cob pigmentation in an epiallele of *P1-wr* called P1-wr*. Therefore, the disruption of this region in *P1-mm* may lead to suppression of cob pigmentation that is typical of most *P1-mm* ears. Our *P1-mm* phenotypic association data also showed that the absence of cob pigmentation was strictly related with the mosaic suppression of pericarp pigmentation. Thus, in addition to cob pigmentation, the structural difference in the intron 2 region could have engendered colorless sectors on *P1-mm* pericarp. The mosaic pericarp pigmentation in *P1-mm* may reflect variable activity of a suppression mechanism associated with the presence of multiple gene copies.

### Competing Transcriptional Enhancing and Suppressing Epigenetic Mechanisms May Be Responsible for the Unique Expression Patterns of *P1-mm*

Nearly 30 years ago, Drew Schwartz proposed a presetting and erasure model for the expression of pigmentation in *P1-vv* [50]. Pigmentation in the kernel gown was immediately preset to a suppressed state; however the suppression on the kernel crown and cob glumes was not set until the subsequent generation. The suppressed state of the kernel gown in *P1-vv*, could usually be erased to a *P1-rr* state if progeny were sown from kernels exhibiting full crown pigmentation. Based on these observations, Schwartz hypothesized that the *p1* gene contains distinct cis-regulatory regions for kernel crown, kernel gown, and cob glumes pigmentation. We now know that such temporal, non heritable and tissue-specific differences in expression can all be explained by changes to the epigenetic state of a gene. More recent studies have begun to shed light on how the epigenetic state of specific regulatory regions of *p1* correlates with allelic expression differences.

Herein, we found that DNA methylation levels did not correlate with pericarp pigmentation levels of *P1-mm*. We showed that *P1-mm* plants with pigmented, colorless, or self-red revertant pericarps exhibited no difference in DNA methylation. However, the extensive DNA methylation differences between *P1-wr* and *P1-mm* would be predicted to affect their respective chromatin structures. In other words, the DNA methylation status of *P1-mm* may report for relatively unstable chromatin structure. Relative to *P1-wr, P1-mm* was shown to be both hypermethylated and hypomethylated, depending on which region was assayed. The DNA of *P1-mm* was hypermethylated downstream from the transcription start site, but was devoid of methylation at a distal floral organ enhancer. The opposing DNA methylation modifications at these distinct regulatory regions may respectively direct the placement of *P1-mm* in euchromatin and heterochromatin. Thus, for unstable *P1-mm* alleles, epigenetic enhancing and suppressing mechanisms may compete to dictate the outcome of gene expression and pigmentation.

The aforementioned hypothesis for the competition between *cis* regulatory regions is strengthened when the regions exhibiting DNA methylation differences in *P1-mm* are taken into account. In several studies, elevated *P1-wr* expression that results in pericarp pigmentation is correlated with the hypomethylation at a distal floral organ enhancer. For example, several *P1-wr* alleles that have pericarp pigmentation confined to the kernel gown are partially hypomethylated at this enhancer [10], [51], [52]. Additionally, both *P1-wr-mum6* and *P1-wr Ufo1* plants that have ectopic pericarp pigmentation also exhibit partial hypomethylation in this region [21], [47]. Thus, DNA hypomethylation at the distal floral organ enhancer has been suggested to be conducive to pericarp pigmentation. It was therefore interesting to discover that the substantial hypomethylation of the distal floral organ enhancer did not always correlate with the presence of variegated pericarp pigmentation in *P1-mm*. Such variability in pigmentation was not explainable until other regions of *P1-mm* were examined for DNA methylation. The DNA hypermethylation downstream from the transcription start site in *P1-mm* parallels results found for the P1-wr* epiallele [49]. In both P1-wr* and *P1-mm* this hypermethylation was correlated with the suppression of cob pigmentation. However, in *P1-mm*, we showed that the suppression of cob pigmentation was correlated with the instability of pericarp pigmentation. Thus, the hypermethylation downstream from the transcription start site may have a suppressing affect on both pericarp and cob glumes pigmentation.

Hypermethylation has typically been associated with heterochromatin and transcriptional suppression, whereas hypomethylation is associated with euchromatin and transcriptional activation [53]. In this model, the hypomethylation at the distal floral organ enhancer would report for the involvement of a mechanism that induces euchromatin formation and gene expression. Meanwhile, the hypermethylation downstream from the transcription start site would suggest for the involvement of a mechanism that induces heterochromatin formation and transcriptional suppression. The interplay between the unique gene structure and DNA methylation may have rendered *P1-mm* a rather recalcitrant target for the heterochromatin machinery. However, it is likely that chromatin states of *P1-mm* can become more stable though the continued selection of either heavy of light pigmentation levels in self pollinated progeny. For example, the *P1-mm-H* allele with dark mosaic pigmentation would be expected to have a more relaxed chromatin structure than the *P1-mm-L* allele that produces light mosaic pigmentation. The self-red revertant alleles may be the outcome of infrequent events, in which the chromatin structure changed so that the aforementioned suppression mechanism is no longer active. We conclude that mosaic pericarp pigmentation of *P1-mm* is likely induced by the absence or modification of some of its tandemly-repeated gene copies that were present in a *p1* ancestral allele. Such structural modifications in *P1-mm* may have led to the variable activity of the epigenetic silencing mechanism that in *P1-wr* stably regulates the suppression in pericarp tissue. This study paves the way for the future cloning of *P1-mm* and the further dissection of the epigenetic mechanism that governs its unique expression patterns.

## Materials and Methods

### *P1-mosaic* Stocks

The mosaic and self-red revertant stocks of *P1-mm* were introgressed into *p1-ww* [4co63] by R.A. Brink [10], [13]. Subsequently, the Brink's collection was maintained by self pollination at the Maize Genetics Cooperation - Stock Center (University of Illinois, Urbana-Champaign). The Brink's stocks are of unique origins (Table 1) with the exception of *P1-mm-CFS-315*, which was derived from the mosaic *P1-mm-CFS-287* allele. In this study, we used *P1-mm* stocks generously given to us by Dr. Thomas Peterson (Iowa State University). These stocks include unstable heavy (*P1-mm-H)* and light (*P1-mm-L*) mosaic stocks that were generated by repeatedly selecting for high and low pigmentation levels in self pollinated progenies (Figure 1B). Additionally, we obtained from Dr. Peterson stable *P1-rr*-like revertants from *P1-mm-H*. These revertants called *P1-mm-7F32* and *P1-mm-542A* had red pericarp and red cob glumes (Figure 1B). The *P1-mm-7F32* stock had red patterned appearance, whereas the *P1-mm-542A* had uniformly red pericarps and cob glumes. An additional *P1-mm* stock, called *P1-mm-d* (derivative), was developed by crossing *P1-mm-H* and *P1-wr* plants. Resulting F_2_ generation plants were crossed with *p1-ww* [4co63] and the ensuing progeny was self pollinated. From these crosses, *P1-mm-d*, *P1-wr*, and *p1-ww* [4co63] segregants were molecularly examined.

### Generation of *P1-mosaic* Stock with Inactive *Mutator* Elements

The effect of *Mutator* (*Mu*) transposon activity on *P1-mm* variegation was tested by crossing a homozygous *P1-mm* stock with a *p1-ww* stock that was heterozygous for *Mu killer* (*Muk*) [31], [32], [33]. The presence of *Muk* was determined by PCR assay available at http://plantbio.berkeley.edu/~mukiller/using.html

### DNA Gel Blot Analysis

Seedling leaf genomic DNA was prepared using a modified CTAB method [54]. PCR genotyping was performed using standard conditions with primers listed in Table S1. Restriction digestion was achieved by using enzymes, reagents and protocols from Promega (Madison, WI). Restricted genomic DNA was fractionated on 0.8% agarose gels and subsequently transferred to Nylon membranes. Membranes were prehybridized for 15 h and hybridized for 15 h at 65°C in buffer containing NaCl (1 M), SDS (1%), Tris-HCl (10 mM) and 0.25 mg/ml salmon sperm DNA [15]. The DNA probes used for hybridizations were labeled with [α-^32^P]dCTP through random priming using Prime-It® RmT Random Primer Labeling Kit (Stratagene, La Jolla, CA). Blots were stripped of previous probe by boiling in 0.1% SDS before they were reused. The relative copy number of *P1-mm* was estimated by comparing its signal intensity with that of other *p1* alleles for which copy number is known [19]. The methylation-sensitive restriction enzymes *Hpa*II, and *Sal*I were used to characterize the DNA methylation status of the *p1* gene at several regions.

### cDNA Sequence Analysis

Pericarp tissue was collected 18 days after pollination and 500 mg was used for RNA extraction. Total RNA was isolated using a standard Trizol-based extraction protocol (Invitrogen Corporation, Carlsbad, CA). 25 µg of total RNA was subsequently incubated with DNAseI according to the manufacturer's directions (Invitrogen Corporation, Carlsbad, CA). First-strand cDNA was synthesized using the SuperScript® III First-Strand Synthesis System (Invitrogen Corporation, Carlsbad, CA). The reverse transcription reaction was done by using an oligo(dT) adaptor primer and 5 µg of DNAseI-treated total RNA. The cDNA was used as a template for PCR reactions with gene specific primers listed in Table S1. PCR products were cloned into a PT7-Blue cloning vector (Novagen) and sequenced. Sequences were aligned using the ClustalW2 web-based program.

### *p1* Probe Fragments

The *p1* probe fragments F8B, F8C, F13, and F15 used in this study have previously been described [19], [49], [55]. The *p1* probe fragment 8D was purified from the PCR product amplified using the WR8F and WR7R primers (Table S1).

### Genomic Bisulfite Sequencing

Two plants each of three *P1-mm* alleles and *P1-wr* [W23] were subjected to genomic bisulfite sequencing. Eight micrograms seedling leaf DNA from individual plants was restricted with *Ssp*I and *Sca*I and purified with phenol-chloroform before treating with sodium bisulfite [49], [56]. The upper strand of the sodium bisulfite treated DNA was amplified using specially designed PCR primers [47] to yield a 439 bp band from the *p1* distal enhancer (positions -5104 to -4666 of EF165349). The length of the region studied in *P1-mm* alleles (*P1-mm-d*, *P1-mm-542A*, and *P1-mm-7F32*) is 443 bp due to a four bp insertion (at position -5088) in these alleles. Furthermore, due to the insertion, a CNG site becomes a CNN site in these alleles. PCR products were gel-purified and cloned with a TOPO TA cloning kit (Invitrogen Corporation, Carlsbad, CA) and sequenced using vector primers.

### Real-Time Quantitative PCR

Genomic DNA was quantified using NanoDrop (NanoDrop 1000, Thermo Scientific) and diluted to the same concentration (100 ng/µl) for Real-Time PCR use according to the NanoDrop measurements. The DNA was subsequently run on agarose gel to verify its quality and concentration. RT-qPCR was performed in the ABI7500 fast real-time PCR system, using SYBR Green I (Roche, Indianapolis, IN) as the detection system and the default program: 10 minutes of pre-incubation at 95°C followed by 40 cycles for 15 seconds at 95°C and one minute at 60°C. Each RT-qPCR reaction was carried out in 20 µl volumes containing 100 ng of genomic DNA from each genotype, 1 µM RTF8B-1 primers and 2X SYBR Green I master mix. Real-time PCR oligonucleotide primers which located on the *p1* F8B fragment are designed to amplify a 60 bp fragment (positions 8299–8359 of EF165349) using Primer Express 3.0 (Applied Biosystems). The ΔCt was calculated as follows ΔCt = Ct (*P1-rr-4B2*, *P1-wr* or *P1-mm*)-Ct (*P1-rr*) for each genotype. *P1-rr* served as a reference genotype because it is known to have one gene copy. Hence, the results for copy number are expressed in N-fold (N = 2^−ΔCt^) changes relative to *P1-rr-4B2*. Standard errors were calculated using three independent preparations of genomic DNA and three technical replicates for each genomic DNA preparation.

## Supporting Information

Figure S1 Alignment of 5′ and 3′ cDNA sequences obtained form three *P1-mm* alleles with known sequences of *P1-wr*, *P1-rr*, and *p2*.
(0.04 MB DOC)

Table S1 Sequences of PCR primers used in this study.
(0.04 MB DOC)

## References

[1] Coe EH (2001). The origins of maize genetics.. Nat Rev Genet.

[2] Correns CG (1900). Mendel's Regel uber das Verhalten der Nachkommenschaft der Rassenbastarde.. Berichte Deut Bot Ges.

[3] Emerson RA (1911). Genetic correlation and spurious allelomorphism in maize.. Ann Rept Nebraska Agric Exp Sta.

[4] Anderson EG (1924). Pericarp studies in maize: II. The allelomorphism of a series of factors for pericarp color.. Genetics.

[5] Emerson RA (1914). The inheritance of a recurring somatic variation in variegated ears of maize.. Am Nat.

[6] Emerson RA (1917). Genetic studies of variegated pericarp in maize.. Genetics.

[7] Hayes HK (1917). Inheritance of a Mosaic Pericarp Pattern Color of Maize.. Genetics.

[8] Anderson EG, Ter Louw AL (1929). Description of Mosaic Pericarp Color in Maize.. Mich Acad Sci Arts and Letters Papers.

[9] Eyster WH (1925). Mosaic Pericarp in Maize.. Genetics.

[10] Brink RA, Styles ED (1966). A collection of pericarp factors.. Maize Genetics Cooperation Newsletter.

[11] Suto T (1952). Genetic analysis of a mosaic pericarp in maize, with special reference to the genetic change of the color pattern induced by the possiby unequal crossing-over in a small regoin containing the *P-*locus and its neighbors of chromosome I.. Journal of the Faculty of Science, Hokkaido University.

[12] Fedoroff N (1998). Marcus Rhoades and transposition.. Genetics.

[13] Barclay PC, Brink RA (1954). The Relation between Modulator and Activator in Maize.. Proc Natl Acad Sci U S A.

[14] Athma P, Grotewold E, Peterson T (1992). Insertional mutagenesis of the maize P gene by intragenic transposition of Ac.. Genetics.

[15] Athma P, Peterson T (1991). Ac induces homologous recombination at the maize P locus.. Genetics.

[16] Grotewold E, Athma P, Peterson T (1991). A possible hot spot for *Ac* insertion in the maize *P* gene.. Mol Gen Genet.

[17] Zhang F, Peterson T (2005). Comparisons of maize pericarp color1 alleles reveal paralogous gene recombination and an organ-specific enhancer region.. Plant Cell.

[18] Zhang F, Peterson T (2006). Gene conversion between direct noncoding repeats promotes genetic and phenotypic diversity at a regulatory locus of Zea mays (L.).. Genetics.

[19] Chopra S, Athma P, Li XG, Peterson T (1998). A maize Myb homolog is encoded by a multicopy gene complex.. Mol Gen Genet.

[20] Goettel W, Messing J (2009). Change of gene structure and function by non-homologous end-joining, homologous recombination, and transposition of DNA.. PLoS Genet.

[21] Chopra S, Cocciolone SM, Bushman S, Sangar V, McMullen MD (2003). The maize *Unstable factor for orange1* is a dominant epigenetic modifier of a tissue specifically silent allele of *pericarp color1*.. Genetics.

[22] Zhang P, Chopra S, Peterson T (2000). A segmental gene duplication generated differentially expressed myb-homologous genes in maize.. Plant Cell.

[23] Sidorenko LV, Li X, Cocciolone SM, Chopra S, Tagliani L (2000). Complex structure of a maize Myb gene promoter: functional analysis in transgenic plants.. Plant J.

[24] Sidorenko LV, Peterson T (2001). Transgene-induced silencing identifies sequences involved in the establishment of paramutation of the maize p1 gene.. Plant Cell.

[25] Sekhon RS, Chopra S (2009). Progressive Loss of DNA Methylation Releases Epigenetic Gene Silencing From a Tandemly Repeated Maize Myb Gene.. Genetics.

[26] Emerson RA (1939). A Zygotic Lethal in Chromosome 1 of Maize and Its Linkage with Neighboring Genes.. Genetics.

[27] Buckler ES, Gaut BS, McMullen MD (2006). Molecular and functional diversity of maize.. Curr Opin Plant Biol.

[28] Brawn RI (1966). A test for Spm control of mosaic pericarp.. Maize Genetics Cooperation Newsletter.

[29] Brawn (1967). Spm regulation of Diffuse and mosaic pericarp..

[30] Nelson OE (1991). Mosaic pericarp does not result from an Spm insertion.. Maize Genetics Cooperation Newsletter.

[31] Slotkin RK, Freeling M, Lisch D (2003). Mu killer causes the heritable inactivation of the Mutator family of transposable elements in Zea mays.. Genetics.

[32] Slotkin RK, Freeling M, Lisch D (2005). Heritable transposon silencing initiated by a naturally occurring transposon inverted duplication.. Nat Genet.

[33] Lisch D, Carey CC, Dorweiler JE, Chandler VL (2002). A mutation that prevents paramutation in maize also reverses Mutator transposon methylation and silencing.. Proc Natl Acad Sci U S A.

[34] Zhang F, Peterson T (2005). Comparisons of maize *pericarp color1* alleles aeveal paralogous gene recombination and an organ-specific enhancer region.. Plant Cell.

[35] Li G, Hall TC, Holmes-Davis R (2002). Plant chromatin: development and gene control.. Bioessays.

[36] Bender J (1998). Cytosine methylation of repeated sequences in eukaryotes: the role of DNA pairing.. Trends Biochem Sci.

[37] Henikoff S (1998). Conspiracy of silence among repeated transgenes.. Bioessays.

[38] Assaad FF, Tucker KL, Signer ER (1993). Epigenetic repeat-induced gene silencing (RIGS) in Arabidopsis.. Plant Mol Biol.

[39] Ye F, Signer ER (1996). RIGS (repeat-induced gene silencing) in Arabidopsis is transcriptional and alters chromatin configuration.. Proc Natl Acad Sci U S A.

[40] Schotta G, Ebert A, Dorn R, Reuter G (2003). Position-effect variegation and the genetic dissection of chromatin regulation in Drosophila.. Semin Cell Dev Biol.

[41] Sabl JF, Henikoff S (1996). Copy number and orientation determine the susceptibility of a gene to silencing by nearby heterochromatin in Drosophila.. Genetics.

[42] Chandler VL (2004). Poetry of b1 paramutation: cis- and trans-chromatin communication.. Cold Spring Harb Symp Quant Biol.

[43] Matzke M, Mette MF, Jakowitsch J, Kanno T, Moscone EA (2001). A test for transvection in plants: DNA pairing may lead to trans-activation or silencing of complex heteroalleles in tobacco.. Genetics.

[44] Grant-Downton RT, Dickinson HG (2004). Plants, pairing and phenotypes–two's company?. Trends Genet.

[45] Watanabe K, Pecinka A, Meister A, Schubert I, Lam E (2005). DNA hypomethylation reduces homologous pairing of inserted tandem repeat arrays in somatic nuclei of Arabidopsis thaliana.. Plant J.

[46] Bender J (2004). Chromatin-based silencing mechanisms.. Curr Opin Plant Biol.

[47] Robbins ML, Sekhon RS, Meeley R, Chopra S (2008). A Mutator Transposon Insertion Is Associated With Ectopic Expression of a Tandemly Repeated Multicopy Myb Gene pericarp color1 of Maize.. Genetics.

[48] Piffanelli P, Ramsay L, Waugh R, Benabdelmouna A, D'Hont A (2004). A barley cultivation-associated polymorphism conveys resistance to powdery mildew.. Nature.

[49] Sekhon RS, Peterson T, Chopra S (2007). Epigenetic modifications of distinct sequences of the p1 regulatory gene specify tissue-specific expression patterns in maize.. Genetics.

[50] Schwartz D (1982). Tissue-specific regulation of gene function: Presetting and erasure.. Proc Natl Acad Sci U S A.

[51] Cocciolone SM, Chopra S, Flint-Garcia SA, McMullen MD, Peterson T (2001). Tissue-specific patterns of a maize Myb transcription factor are epigenetically regulated.. Plant J.

[52] Robbins ML, Peterson T, Chopra S (2008). Identification and characterizations of *P1-wr* epialleles in maize that show a gain of pericarp function.. Maize Genet Coop Newslett.

[53] Gehring M, Henikoff S (2007). DNA methylation dynamics in plant genomes.. Biochim Biophys Acta.

[54] Saghai-Maroof MA, Soliman KM, Jorgensen RA, Allard RW (1984). Ribosomal DNA spacer-length polymorphisms in barley: mendelian inheritance, chromosomal location, and population dynamics.. Proc Natl Acad Sci U S A.

[55] Lechelt C, Peterson T, Laird A, Chen J, Dellaporta SL (1989). Isolation and molecular analysis of the maize P locus.. Mol Gen Genet.

[56] Jacobsen SE, Sakai H, Finnegan EJ, Cao X, Meyerowitz EM (2000). Ectopic hypermethylation of flower-specific genes in Arabidopsis.. Curr Biol.

