# Supplementary material for: Gene Structure Induced Epigenetic Modifications of pericarp color1 Alleles of Maize Result in Tissue-Specific Mosaicism
Source: PLoS One. 2009 Dec 14;4(12):e8231. doi: 10.1371/journal.pone.0008231 (PMC2788268; doi:10.1371/journal.pone.0008231)
Supplement: Figure S1 — Alignment of 5′ and 3′ cDNA sequences obtained form three P1-mm alleles with known sequences of P1-wr, P1-rr, and p2. (0.04 MB DOC) [file pone.0008231.s001.doc]

Figure S1.

5’ cDNA alignment of *P1-mosiac* alleles with *P1-wr, P1-rr*, and *p2*

*P1-mm-L* ACGCGCGACCAGCTGCTAACC---------GTGCGCAAGTAGTAGTGCGACTTCGCCGCC 51

*P1-mm-H* ACGCGCGACCAGCTGCTAACC---------GTGCGCAAGTAGTAGTGCGACTTCGCCGCC 51

*P1-mm-542A* ACGCGCGACCAGCTGCTAACC---------GTGCGCAAGTAGTAGTGCGACTTCGCCGCC 51

*P1-wr* ACGCGCGACCAGCTGCTAACC---------GTGCGCAAGTAGTAGTGCGACTTCGCCGCC 51

*P1-rr* ACGCGCGACCAGCTGCTAACCCTGCTAACCGTGCGCAAGTAGTAGTGCGACTTCGCCGCC 60

*p2*  ------------------------------------------------------------

*P1-mm-L* GGCCGGGATCGCTAGCTCGATCGATCG-GCGGGACCACATACGACTCCGGTGTGGCCAGC 110

*P1-mm-H* GGCCGGGATCGCTAGCTCGATCGATCG-GCGGGACCACATACGACTCCGGTGTGGCCAGC 110

*P1-mm-542A* GGCCGGGATCGCTAGCTCGATCGATCG-GCGGGACCACATACGACTCCGGTGTGGCCAGC 110

*P1-wr* GGCCGGGATCGCTAGCTCGATCGATCG-GCGGGACCACATACGACTCCGGTGTGGCCAGC 110

*P1-rr* GGCCGGGATCGCTAGCTCGATCGATCG-GCGGGACCACATACGACTCCGGTGTGGCCAGC 119

*p2*  ----------------ACGCACGCGCGCACGCGACCAGCTGCTAGCCGTGCGCAAGTAGT 44

** ** ** ** ***** * * * * * * **

*P1-mm-L* GGCGGCCGGGCCGGGGAACGCACGTGCTGCGAGCGAGCGAGGGCAGACGCTAGCTGTTGC 170

*P1-mm-H* GGCGGCCGGGCCGGGGAACGCACGTGCTGCGAGCGAGCGAGGGCAGACGCTAGCTGTTGC 170

*P1-mm-542A* GGCGGCCGGGCCGGGGAACGCACGTGCTGCGAGCGAGCGAGGGCAGACGCTAGCTGTTGC 170

*P1-wr* GGCGGCCGGGCCGGGGAACGCACGTGCTGCGAGCGAGCGAGGGCAGACGCTAGCTGTTGC 170

*P1-rr* GGCGGCCGGGCCGGGGAACGCACGTGCTGCGAGCGAGCGAGGGCAGACGCTAGCTGTTGC 179

*p2*  G-CGAGCTCGCCGCCG---GCCAGGGTCGCTAGCT--CGATTGGAGGGACCAGCTGATAC 98

* ** * **** * ** * * ** *** *** * ** * ***** * *

*P1-mm-L* CGGGAGCTAGCCGGCGCGCGATGGGGAGGGCGCCGTGCTGCGAGAAGGTGGGGCTCAAGC 230

*P1-mm-H* CGGGAGCTAGCCGGCGCGCGATGGGGAGGGCGCCGTGCTGCGAGAAGGTGGGGCTCAAGC 230

*P1-mm-542A* CGGGAGCTAGCCGGCGCGCGATGGGGAGGGCGCCGTGCTGCGAGAAGGTGGGGCTCAAGC 230

*P1-wr* CGGGAGCTAGCCGGCGCGCGATGGGGAGGGCGCCGTGCTGCGAGAAGGTGGGGCTCAAGC 230

*P1-rr* CGGGAGCTAGCCGGCGCGCGATGGGGAGGACGCCGTGCTGCGAGAAGGTGGGGCTCAAGC 239

*p2*  -GACTCCGGTGTGGCGCGCGATGGGGAGGGCGCCGTGCTGCGAGAAGGTGGGGCTCAAGC 157

* * ***************** ******************************

*P1-mm-L* GAGGGAGGTGGACGGCGGAAGAGGACCAGTTACTTGCCAACTACATTGCGGAGCACGGCG 290

*P1-mm-H* GAGGGAGGTGGACGGCGGAAGAGGACCAGTTACTTGCCAACTACATTGCGGAGCACGGCG 290

*P1-mm-542A* GAGGGAGGTGGACGGCGGAAGAGGACCAGTTACTTGCCAACTACATTGCGGAGCACGGCG 290

*P1-wr* GAGGGAGGTGGACGGCGGAAGAGGACCAGTTACTTGCCAACTACATTGCGGAGCACGGCG 290

*P1-rr* GAGGGAGGTGGACGGCGGAAGAGGACCAGTTACTTGCCAACTACATTGCGGAGCACGGCG 299

*p2*  GAGGGAGGTGGACGGCGGAGGAGGACCAGTTACTTGCCAACTACATTGCGGAGCACGGCG 217

******************* ****************************************

*P1-mm-L* AGGGGTCCTGGAGGTCGCTGCCCAAGAATGCAGGCCTGCTCCGGTGCGGCAAGAGCTGCC 350

*P1-mm-H* AGGGGTCCTGGAGGTCGCTGCCCAAGAATGCAGGCCTGCTCCGGTGCGGCAAGAGCTGCC 350

*P1-mm-542A* AGGGGTCCTGGAGGTCGCTGCCCAAGAATGCAGGCCTGCTCCGGTGCGGCAAGAGCTGCC 350

*P1-wr* AGGGGTCCTGGAGGTCGCTGCCCAAGAATGCAGGCCTGCTCCGGTGCGGCAAGAGCTGCC 350

*P1-rr* AGGGGTCCTGGAGGTCGCTGCCCAAGAATGCAGGCCTGCTCCGGTGCGGCAAGAGCTGCC 359

*p2*  AGGGGTCCTGGAGGTCGCTGCCCAAGAATGCAGGCCTGCTCCGGTGCGGCAAGAGCTGCC 277

************************************************************

*P1-mm-L* GGCTCCGGTGGATCAACTACCTTCGGG 377

*P1-mm-H* GGCTCCGGTGGATCAACTACCTTCGGG 377

*P1-mm-542A* GGCTCCGGTGGATCAACTACCTTCGGG 377

*P1-wr* GGCTCCGGTGGATCAACTACCTTCGGG 377

*P1-rr* GGCTCCGGTGGATCAACTACCTTCGGG 386

*p2* GGCTCCGGTGGATCAACTACCTCCGGG 304

********************** ****

3’ cDNA alignment of *P1-mosiac* alleles with *P1-wr, P1-rr*, and *p2*

*P1-mm-L* GACGATCGCGAGCTGGAGGCGTTCGAGACTTGGCTCCTGTCCGACTCGTTCTGACGGCTC 60

*P1-mm-H* GACGATCGCGAGCTGGAGGCGTTCGAGACTTGGCTCCTGTCCGACTCGTTCTGACGGCTC 60

*P1-mm-542A* GACGATCGCGAGCTGGAGGCGTTCGAGACTTGGCTCCTGTCCGACTCGTTCTGACGGCTC 60

*P1-wr* GACGATCGCGAGCTGGAGGCGTTCGAGACTTGGCTCCTGTCCGACTCGTTCTGACGGCTC 60

*P1-rr* GACGATCGCGAGCTGGAGGCGTTCGAGACTTGGCTCCTGTCCGACTCGTTCTGACGGCTC 60

*p2*  GACGATCGCGAGCTGGAGGCGTTCGAGACTTGGCTCCTGTCCGACTCGTTCTGACGGCTC 60

************************************************************

*P1-mm-L* CGGTCACCGGACCGATCAGACAGAC--------CAACCAAGGTG-GCCCGGCCATATGGT 111

*P1-mm-H* CGGTCCCCGGACCGATCAGACAGAC--------CAACCAAGGTG-GCCCGGCCATATGGT 111

*P1-mm-542A* CGGTCACCGGACCGATCAGACAGAC--------CAACCAAGGTG-GCCCGGCCATATGGT 111

*P1-wr* CGGTCACCGGACCGATCAGACAGAC--------CAACCAAGGTG-GCCCGGCCATATGGT 111

*P1-rr* CGGTCACCGGACCGATCAGACAGACCAAATAATTGGGTCACGTGTGCTCGCTCGCTCGCT 120

*p2*  CGGTCATCGGACCGATCAGACAGACAGACAGACCAACCAAGGTG-GCCCGGCCATATGGT 119

***** ****************** * *** ** ** * * *

*P1-mm-L* CGACGCCGCTA-GTAGGCGTTG-CTCGTGTGTACAGTTTTTTTTTCTTTT--TT----GT 163

*P1-mm-H* CGACGCCGCTA-GTAGGCGTTG-CTCGTGTGTACAGTTTTTTTTTCTTTT--TT----GT 163

*P1-mm-542A* CGACGCCGCTA-GTAGGCGTTG-CTCGTGTGTACAATTTTTTTTTCTTTT--TT----GT 163

*P1-wr* CGACGCCGCTA-GTAGGCGTTG-CTCGTGTGTACAGTTTTTTTTTCTTTT--TT----GT 163

*P1-rr* CGCTGCCGTCGCGTGGGTCTTGGTTCAGATGGCCAAATAATTGGGAAAAAAATTCTACGC 180

*p2* CGACGCCGCTA-GTAGGCGTTG-CTCGTGTGTACAGTTTTTTTTTCTTTT--TTTATTGT 175

** **** ** ** *** ** ** ** * ** ** *

*P1-mm-L* AGATTTTGTTCTTAGCTCTTGCTG--------TTTCGTGTATG--------GCATGTAGA 207

*P1-mm-H* AGATTTTGTTCTTAGCTCTTGCTG--------TTTCGTGTATG--------GCATGTAGA 207

*P1-mm-542A* AGATTTTGTTCTTAGCTCTTGCTG--------TTTCGTGTATG--------GCATGTAGA 207

*P1-wr* AGATTTTGTTCTTAGCTCTTGCTG--------TTTCGTGTATG--------GCATGTAGA 207

*P1-rr* GGCAGGGCCGTAAAGCCACCACCG---------TGCGCTCCTGATGTCGATGCCTGCCGC 231

*p2* AGATGTTGTTCTTAGCTCGTGCTGACGTGCTGTTTCGTGTATGACGTACTGGCATGTAGA 235

* *** * * * ** ** ** ** *

*P1-mm-L* ATAGAGAGCAGAGAGTCGTATGGCAT----TGTAAAGGTAGTTCTCCC-GCCGGTGAAGT 262

*P1-mm-H* ATAGAGAGCAGAGAGTCGTATGGCAT----TGTAAAGGTAGTTCTCCC-GCCGGTGAAGT 262

*P1-mm-542A* ATAGAGAGCAGAGAGTCGTATGGCAT----TGTAAAGGTAGTTCTCCC-GCCGGTGAAGT 262

*P1-wr* ATAGAGAGCAGAGAGTCGTATGGCAT----TGTAAAGGTAGTTCTCCC-GCCGGTGAAGT 262

*P1-rr* GTGGAGCTCTTGCGTATCTAACGCTCC---CACGACAATCACCCTTCCAGACGGCTCGAA 288

*p2*  ATAGAGAGCAGAGAGTCGTATGGATTGGCGTGTAAAGGTAGTTCTCCC-GCCGGTGAAGT 294

* *** * ** * * * ** ** * ***

*P1-mm-L* GAAGACCAATAAAAAAAAGCAGCGCAGCAGTAGGCGCTGCTGCAGCCTGCAC--GGCGGC 320

*P1-mm-H* GAAGACCAATAAAAAAAAGCAGCGCAGCAGTAGGCGCTGCTGCAGCCTGCAC--GGCGGC 320

*P1-mm-542A* GAAGACCAATAAAAAAAAGCAGCGCAGCAGTAGGCGCTGCTGCAGCCTGCAC--GGCGGC 320

*P1-wr* GAAGACCAATAAAAAAAAGCAGCGCAGCAGTAGGCGCTGCTGCAGCCTGCAC--GGCGGC 320

*P1-rr* TTACATACGACAGGATCGGCTCCGCTCTACTCCGTTCTGTTCGCTTCTGCTTTAGGTGCG 348

*p2*  GAAGAC------------------------------------------------------ 300

* *

*P1-mm-L* GTTTTGGCA------------------------- 329

*P1-mm-H* GTTTTGGCAGCGCGTGCTTTGGCTTTTTGTG--- 351

*P1-mm-542A* GTTTTGGCAGCGCGTGCTTTGGCTTTTTGTGAAC 354

*P1-wr* GTTTTGGCA------------------------- 329

*P1-rr* TGCCTAGCAGATGGTGAGGCGGCGTCGCGCGGC- 381

*p2*  ----------------------------------
