# Supplementary material for: Gene Structure Induced Epigenetic Modifications of pericarp color1 Alleles of Maize Result in Tissue-Specific Mosaicism
Source: PLoS One. 2009 Dec 14;4(12):e8231. doi: 10.1371/journal.pone.0008231 (PMC2788268; doi:10.1371/journal.pone.0008231)
Supplement: Table S1 — Sequences of PCR primers used in this study. (0.04 MB DOC) [file pone.0008231.s002.doc]

**Table S1:** Sequences of PCR primers used in this study.

| Name | Sequence | Purpose | *P1*  Alleles Amplified | Pdt. Size (bp) |
| --- | --- | --- | --- | --- |
| MRF | 5’TGGAGCTCTTGCGTATCTAACGCT 3’ | Genotyping | *P1‑vv, P1‑rr‑4B2,*  *P1-rw1077* | 481 |
| MRR | 5’ AGTGTGCACAGGGACACTTGAGTA 3’ |
| WRJ | 5’ CTGTCGGCTACTATCCCCTTGGTGA 3’ | Genotyping | *P1‑mm,*  *P1-wr*, *P1‑rw1077* | 618 |
| WRK | 5’ GATCGCGAGCTGGAGGCGTTCGAGAC 3’ |
| RTF8B-1F | 5’ CAGTCCGGTCCTTGTCAGTCA 3 | Real Time qPCR | *P1‑mm,*  *P1-wr*, *P1‑rr4B2* | 60 |
| RTF8B-1R | 5’ CACACGTACGGGCAACTTCA 3’ |
| EP5-8 | 5’ACGCACGCGCGACCAGCTGCTAACCGTG3’ | RT-PCR | *P1-mm* | 380 |
| EP3-13 | 5’AGGAATTCCGCCCGAAGGTAGTTGATCC3’ |
| EP5-16 | 5’ GACGATCGCGAGCTGG 3’ | RT-PCR | *P1-mm* | 351 |
| adapter primer | 5’ GCCTCGAGAATTCAAGCTT 3’ |
| oligodT-adapter | 5’GCCTCGAGAATTCAAGC T17 | cDNA synthesis | *P1-mm* | - |
| WR8F | 5’ATGAACCAAACAGGCTCGAT 3’ | 8D probe | *-* | 2249 |
| WR7R | 5’ CTTCCCTGTTGCCTTGATC 3’ |
